# Targeting immunometabolism to treat COVID-19

**DOI:** 10.1093/immadv/ltab013

**Published:** 2021-06-02

**Authors:** Shane Myles O’Carroll, Luke A J O’Neill

**Affiliations:** School of Biochemistry and immunology, Trinity Biomedical Sciences Institute, Trinity College Dublin, Dublin, Ireland

**Keywords:** COVID-19, SARS-CoV-2, Macrophage, Immunometabolism, innate immune reprogramming

## Abstract

The COVID-19 crisis has emphasised the need for antiviral therapies to combat current and future viral zoonoses. Recent studies have shown that immune cells such as macrophages are the main contributors to the inflammatory response seen in the later inflammatory phase of COVID-19. Immune cells in the context of a viral infection such as SARS-CoV-2 undergo metabolic reprogramming to elicit these pro-inflammatory effector functions. The evidence of metabolic reprogramming in COVID-19 offers opportunities for metabolites with immunomodulatory properties to be investigated as potential therapies to combat this inflammatory response. Recent research indicates that the metabolite itaconate, previously known to be broadly antibacterial, may have both antiviral and immunomodulatory potential. Furthermore, low itaconate levels have shown to correlate with COVID-19 disease severity, potentially implicating its importance in the disease. The antiviral potential of itaconate has encouraged researchers to synthesise itaconate derivatives for antiviral screening, with some encouraging results. This review summarises the antiviral and immunomodulatory potential of immunometabolic modulators including metformin, PPAR agonists and TEPP-46 as well as itaconate, and its derivatives and their potential use as a broad spectrum anti-viral agents.

## Introduction:

The innate immune system is our immune system's first line of defence against viruses, such as SARS-CoV-2. It consists of a set of germline-encoded pathogen recognition receptors (PRRs) that function to recognise pathogen-associated molecular patterns (PAMPS). For viral recognition, these PRRs consist of toll-like receptors (TLRs), RIG-I-like receptors (RLRs), NOD-like receptors (NLRs), and DNA sensors. Each sense conserved molecular structures in viruses (largely nucleic acid-based) and induce intracellular signalling cascades which converge on transcription factor activation and subsequent transcription of genes encoding pro-inflammatory cytokines, chemokines, and type 1 interferons to regulate the immune response, induce inflammation and limit viral replication (1).

Apart from the well-known innate immune signalling triggered by a viral infection, metabolic change also occurs downstream of some of these sensors. This was first discovered in response to LPS from gram-negative bacteria. LPS activation of toll-like receptor 4 pathway upregulates aerobic glycolysis and fatty acid synthesis with a concomitant reduction in mitochondrial respiration and β-oxidation in macrophages. Breakpoints occur in the tricarboxylic acid cycle (TCA) cycle after succinate and citrate, causing these metabolites to accumulate. The accumulation of citrate drives mitochondrial ROS (mtROS), nitric oxide accumulation, itaconate production and contributes to the pro-inflammatory phenotype of M1-like macrophages (2). Citrate accumulation also causes acetyl-CoA to build up and contributes to epigenetic changes such as histone acetylation, facilitating transcription of inflammatory genes (3).

Accumulation of succinate drives Warburg-like metabolism and acts by inhibiting prolyl hydroxylases, thus increasing hypoxia-inducible factor 1-alpha (HIF1α) stabilisation. This drives glycolysis and also increases transcription of the pro-inflammatory cytokine interleukin 1 β (IL-1β). Succinate accumulation also causes reverse electron transport (RET) causing mtROS production and the stabilisation of HIF1α (4, 5).

During viral infection the host undergoes two phases of response, the first phase is a pro-inflammatory viral limiting response and the second phase is the resolution and anti-inflammatory phase. Both phases are important to effective viral clearance and host recovery from infection, and mal-adaptive responses in either phase can result in pathologies not directly related to viral load or viral effects, but the immunopathology caused by cells of the immune system (6).

Given the importance of macrophages in both phases of the immune response and the demonstrated metabolic flexibility modulating macrophage phenotype this review focuses on the metabolic reprogramming and immunological response to metabolic modulation and metabolites as a potential therapeutic avenue to the treatment of the pathogenic inflammation associated with COVID-19.

### Metabolic reprogramming during viral infection

Much of the understanding of metabolic reprogramming in innate immunity stems from stimulus with bacterial derived LPS in macrophages. However, there is an increasing appreciation for metabolic change in response to viral sensing. Metabolic reprogramming in host cells in response to viral infection facilitates an effective antiviral innate immune response. Many viruses, however, "hijack" host cell metabolism in an immune evasion strategy. This is done by causing metabolic dysfunction, increasing flux through the pentose phosphate pathway (PPP) to increase nucleotide biosynthesis and diverting amino acid metabolism for virion replication, maturation and viral dissemination (7). Many viruses upregulate PPP by increasing aerobic glycolysis. Such experiments include influenza A infection of mammalian cell-lines, hepatitis C (HCV) infection of hepatocyte cell-lines, adenovirus, Kaposi's sarcoma-associated herpesvirus infection of lymphatic endothelial cells, and Epstein-Barr virus infection of neural progenitor cells (7). Viruses often upregulate glycolysis via viral proteins which interact with and regulate key enzymes. In hepatocyte cell-lines, Non-Structural Protein 5A (NS5A) from HCV binds and activates hexokinase, a rate-limiting glycolytic enzyme, to increase glycolytic rate (8). Creating this Warburg-like metabolism also contributes to the production of type 1 interferons and inflammatory cytokines which aid in the anti-viral immune response (9).

Viral infection often activates HIF1α which contributes to anti-viral innate immunity by producing inflammatory mediators. However, HIF1α also upregulates glycolytic genes needed for virion replication and therefore benefits the virus (10). Many viruses also modulate other metabolic pathways such as downregulating mitochondrial β-oxidation and upregulating fatty acid synthesis to aid virus envelopment for the release of the virus (11).

## Metabolic reprogramming in COVID19 contributes to the antiviral immune response

The progression from SARS-CoV-2 infection to severe COVID-19 is caused by a mal-adaptive immune response which is defined by failure to elicit a timely and robust type-1 interferon innate immune response. This leads to increased viral load, followed by a hyperinflammatory immune response, such as acute phase reactants and inflammatory markers such as interleukin 6 (IL-6), Tumor necrosis factor (TNF), IL1β, and interleukin 18 (IL-18) (12). This bi-phasic nature of COVID-19 progression to severe disease, the early viral phase followed by the inflammatory phase, suggests that anti-inflammatory therapies would be suited to those who present later in the infection and have persistently increased inflammatory markers.

Monocytes and macrophages are the most abundant immune cell-types in SARS-CoV-2-infected lungs and contribute to hyper-inflammatory cytokine production evident in severe COVID-19 (13). SARS-CoV-2 infects many cell types including airway epithelial cells and macrophages. Infection of macrophages causes metabolic reprogramming, similar to that created by LPS, causing an increase in aerobic glycolysis, reduction of the TCA cycle and RET (14). This metabolic reprogramming is mediated by the production of mtROS, stabilisation of HIF1α and HIF1α-mediated transcription of core glycolytic genes involved in glucose import and glycolysis including Glucose transporter 1 (GLUT-1), Pyruvate kinase M2 (PKM2), Lactate dehydrogenase A (LDH-A) and 6-Phosphofructo-2-Kinase/Fructose-2,6-Biphosphatase 3 (PFKFB3). It has also been shown that lactate dehydrogenase, an important enzyme in Warburg metabolism in M1-like macrophages, is a prognostic marker of disease severity in COVID-19 and further highlights the role of glycolysis in COVID-19 (15). Ex-vivo experiments using human monocytes from SARS-CoV-2 infected patients showed that HIF1α protein was significantly increased when compared to uninfected patients. In -vitro experiments with SARS-CoV-2-infected human monocytes elegantly demonstrate that elevated glucose levels and glycolysis facilitates SARS-CoV-2 replication, as blocking glycolysis inhibits viral replication. Furthermore, the increased glycolysis also increases the production of IL-1β which contributes to the hyperinflammatory response of COVID-19. These in-vitro experiments directly implicate HIF1α in viral replication and inflammatory cytokine production as HIF1α inhibition was shown to inhibit SARS-CoV-2 replication and HIF1α activation increased viral replication. Furthermore, HIF1α inhibition also blocked transcription of inflammatory cytokines associated with severe COVID19 including IL1β, TNF, IL6, interferon alpha (IFNα), interferon beta (IFNβ) and Angiotensin-converting enzyme 2 (ACE2) (14). This makes compelling evidence for targeting mtROS-HIFα-

metabolic reprogramming as a potential treatment of severe COVID-19 by inhibiting viral replication and the inflammatory cytokines seen in COVID-19 pathology (14).

## Itaconate and COVID-19

Given these metabolic features, and the discovery that low plasma itaconate levels correlate with COVID-19 disease severity, it seems likely that itaconate might play a role in COVID-19 (16). Itaconate is  a metabolite produced by the decarboxylation of the TCA cycle intermediate cis-aconitate, a reaction catalysed by aconitate decarboxylase 1 (ACOD1), also referred to as immune responsive gene 1 (IRG1) (17, 18). IRG1 and itaconate have been closely associated with the metabolic reprogramming of macrophages in-vitro into an M1-like phenotype as IRG1 is one of the most upregulated genes in classically activated macrophages (19). The main role of itaconate in macrophages is to act in a negative feedback fashion, limiting inflammation (20). Notably, itaconate is also antibacterial against many pathogens including *Salmonella enterica* and *Mycobacterium tuberculosis* (Mtb). These bacteria use carbon sources produced by the glyoxylate shunt to survive during human infection. Itaconate inhibits this glyoxylate shunt via blocking a key enzyme isocitrate lyase. Itaconate also stops Mtb from using carbon sources from cholesterol degradation by inhibiting Mtb mediated detoxification of propionic acid (21).

As well as being anti-bacterial, itaconate is also immunomodulatory. Biochemically, itaconate has been shown to alkylate cystine residues, resulting in changes to protein structure and binding. in-vitro experiments have shown that exogenous itaconate treatment of bone-marrow-derived macrophages upregulates IFN-β in a yet undetermined mechanism (22). Also, itaconate and it's derivative 4-Octyl itaconate, (4-OI), inhibits NOD-, LRR- and pyrin domain-containing protein 3 (NLRP3) inflammasome activation, preventing the secretion of IL-1β (23) which is implicated in COVID-19 immunopathology (24). Furthermore, itaconate reduces other inflammatory cytokines such as IL-6 and interleukin 10 (IL-10) (22) (25).

Not only has itaconate potential immunomodulatory effects, but itaconate has previously been shown to be antiviral against Zika virus (ZIKV) in murine models. ZIKV infection of murine neurons is sensed by the Z-form nucleic acid sensor (ZBP1). ZBP1 causes necroptotic-independent signalling via receptor-interacting protein kinase-1 (RIPK1) and receptor-interacting protein kinase-2 (RIPK2) leading to Interferon Regulatory Factor 1

(IRF1)-dependant upregulation of IRG1 and concomitant itaconate synthesis. In this case, itaconate inhibits succinate dehydrogenase, causing succinate accumulation and driving an antiviral metabolic state in neurons via reduced TCA cycle metabolism and reduced oxygen consumption rate (26). This effect was emphasised by demonstrating that IRG1 knock-out mice have higher levels of ZIKV replication (26). Furthermore, IRG1 mediates an antiviral state in primary cortical neurons against West Nile Virus (WNV), and subsequent knockdown of IRG1 enhanced WNV infection (27). This suggests that itaconate could have antiviral properties against ZIKV, WNV and possibly SARS-CoV-2 as they are all positive-strand RNA viruses.

An interesting link from itaconate to SARS-CoV-2 was recently reported. 4-OI, a derivative of itaconate, has been shown to activate nuclear factor-erythroid factor 2-related factor 2 (NRF2) via the alkylation of Kelch Like ECH Associated Protein 1 (KEAP1)

(28). Furthermore, NRF2 activation is impaired in IRG-1 deficient macrophages treated with LPS. Of note, itaconate has the ability to restore LPS-induced NRF2 activation in IRG-1 deficient macrophages (29, 30). This is interesting as NRF2 functions as a transcription factor that binds to the promoter region of pro-inflammatory genes, preventing RNA Polymerase II binding and also inhibits the transcription of pro-inflammatory cytokines IL6 and IL1β in macrophages (31). NRF2 also drives antioxidant genes which reduce mtROS and HIF1α mediated inflammation. Transcriptome analysis of severe COVID-19 patients has shown that NRF2 dependant genes are suppressed including Heme Oxygenase 1 (HMOX1) and NAD(P)H Quinone Dehydrogenase 1 (NqO1) (32). This suggests that these NRF2 activators could be used as therapies for COVID-19.

Both 4-OI, and dimethyl fumarate (DMF) are both NRF2 activators and inhibit SARS-Cov-2 replication in human airway epithelial cells. This antiviral effect also extends to Zika virus, Herpes Simplex Virus-1 (HSV1), Herpes Simplex Virus -2 (HSV2), and Vaccinia virus (VACV). Furthermore, 4-OI and DMF inhibit the pathogenic interferon response in an NRF2-dependant mechanism by blocking interferon regulatory factor 3 (IRF-3) dimerization. This was shown by demonstrating that  silencing NRF2 rescues IRF-3 signalling. Additionally, NRF2 also reduces interferon responses by reducing stimulator of interferon genes (STING) expression (33). Interestingly, 4-OI and DMF also inhibit SARS-CoV-2

replication in epithelial cells and human peripheral blood mononuclear cells (PBMCs) in a yet undetermined NRF2-independent mechanism.

Importantly, it was also shown that 4-OI and DMF blocked the expression of inflammatory factors including IFNβ1, C-X-C motif chemokine ligand 10 (CXCL-10), TNF, IL-1β, and C-C Motif Chemokine Ligand 5 (CCL5) induced by SARS-CoV-2 infection. Furthermore, pre-treatment with 4-OI or DMF upregulate anti-inflammatory markers such as HMOX1, indicating an increase in the anti-inflammatory responses. This anti-inflammatory effect was also seen in *ex vivo* PBMC samples of severe COVID-19 patients treated with 4-OI. These results show that 4-OI and DMF inhibit SARS-CoV-2 replication in human airway epithelial cells, while also suppressing the macrophage-mediated pathogenic interferon and inflammatory responses seen in the later inflammatory phase of COVID-19 (32).

Other Itaconate derivatives are directly antiviral against Influenza A Virus by binding to the viral nucleoprotein and blocking ribonucleoprotein export from the nucleus to the cytosol and preventing the replication cycle of the virus (34).

## Other metabolic targets in SARS-CoV-2 infection

Mitochondrial dysfunction and the production of mtROS are common features of SARS-CoV-2 infection in human macrophages ex-vivo and in vivo and causes the stabilisation of HIF1α and the following production of pro-inflammatory cytokines (14). This mechanism has also been seen in SARS-CoV-1 studies demonstrating that nucleocapsid protein N disrupts mitochondrial membrane potential and increases mtROS in-vitro (35). Furthermore, SARS-CoV-2 infection of bronchial epithelial cells reduces mitochondrial gene expression, leading to mitochondrial dysfunction and increasing mtROS in-vitro *(36)*. Proteins involved in mitochondrial fission are also suppressed, increasing mitochondrial fusion and which impairs antiviral interferon responses *(37).*

With this in mind, MitoQ is a mitochondria-specific antioxidant that increases autophagy and reduces mtROS in-vitro and in murine models (38). Additionaly, studies demonstrate that MitoQ has antiviral potential. In vivo mouse models have shown that MitoQ reduce dextran sulfate sodium-induced colitis by reducing inflammatory cytokines IL-1β, TNF, and mtROS (39, 40). Furthermore, in-vitro models of Respiratory syncytial virus (RSV) infection of vero and HEK293T-cells have shown that MitoQ  suppresses RSV viral infection by blocking mitochondrial dysfunction and mtROS production (39, 41). Importantly, an in-vitro study using SARS-CoV-2 infection of human monocytes shows that

MitoQ blocks SARS-CoV-2 replication, and reduces HIF1α stabilisation and inflammatory cytokine IL-1β and ACE2 (14)

Increased glycolysis is also associated with SARS-CoV-2 replication. PKM2 is an enzyme that catalyses the final step of glycolysis and also has important roles in immune cell reprogramming and inflammation. However, PKM2 activity depends on its oligomerisation state. PKM2 tetramers are inactive, whereas phosphorylation of PKM2 forms a dimer that enters the nucleus, binds HIF1α, and causes the expression of proinflammatory and glycolytic genes (42). In the context of COVID-19, both PKM2 levels and phosphorylated PKM2 levels increase with COVID-19 severity. This indicates that PKM2 is important in COVID-19 severity via activation of HIF1α and the expression of inflammatory cytokines (43). This metabolic reprogramming causes cytosolic succinate accumulation which activates HIF1α. This is supported by ex-vivo COVID-19 studies show succinate levels and nuclear translocation of both HIF1α and PKM2 positively correlate with COVID-19 disease severity (43). Maintaining PKM2 as a tetramer could therefore be a potential therapy for COVID-19. Compounds such as TEPP-46 maintain PKM2 in a tetramer, inhibiting Warburg-like metabolism, succinate accumulation, inflammatory cytokine IL-1β and inhibits HIF1α dependant transcription of glycolytic genes needed for SARS-CoV-2 (44).

Other potential immunomodulators being proposed for the treatment of COVID-19 include Peroxisome proliferator activated receptor (PPAR) agonists. PPARs are a group of transcription factors which regulate glucose and lipid metabolism as well as macrophage polarisation (45). These transcription factors agonists may be a therapy to dampen the late inflammatory phase of COVID-19 as PPAR-ɣ agonists such as rosiglitazone can block nuclear factor kappa B (NF-κB)-mediated inflammatory cytokine production in lung epithelial cells (46) (47). In the case of COVID-19, lung biopsies of COVID-19 patients show PPAR-ɣ is suppressed in M1-like macrophages, similar to that of PPAR-ɣ knockout macrophages. Furthermore SUMO1, a protein in the PPAR-Ɣ complex was shown to be repressed in severe COVID-19 and shown to interact with a SARS nucleoprotein (48). This suggests that SARS-CoV-2 may interact with SUMO1, repress PPAR-Ɣ activity, leading to the hyperinflammatory response seen in severe COVID-19. PPAR-ɣ agonists could, in theory, be suggested as a therapy to dampen the excessive inflammation seen in the lungs of COVID-19 patients.

## Metformin and DMF in COVID-19

Metformin is a safe, widely used glucose lowering agent used to treat type 2 diabetes mellitus (T2DM). The anti-viral potential of metformin was first discovered in the 1940s when it showed significant anti-influenza properties (49). Metformin has been shown to block ZIKA virus replication, increase innate immune signalling via interferon-stimulated genes such as interferon stimulated gene 15 (ISG15), 2'-5'-Oligoadenylate Synthetase 2 (OAS2) and blocks TNF and CCL5 production in-vitro and in-vivo (50).

Metformin significantly reduced hospital mortality in diabetic patients with COVID-19, suggesting therapeutic potential (51). Metformin's anti-viral effect could be attributed to several potential mechanisms. SARS-CoV-2 entry into cells causes the downregulation of ACE2. ACE2 downregulation dysregulates the rennin-angiotensin system (RAS) which may contribute to increased inflammation. Metformin induces ACE2 expression in Human umbilical vein endothelial cells (HUVEC) cells, potentially preventing RAS dysregulation and reducing inflammation (52). Furthermore, In human endothelial cells, metformin causes increased phosphorylation of ACE2 Ser680 via AMPK. This might suggest that ACE2 phosphorylation might block spike-ACE2 interaction or binding and therefore reduce SARS-CoV-2 cell entry (53). Metformin also blocks IL1β production and increases the anti-inflammatory cytokine IL-10 and inhibits complex 1 in mitochondria, indicating its anti-inflammatory potential for COVID-19 (40). Furthermore, neutrophil extracellular traps (NETs) contribute to the immunopathology seen in COVID-19 (54). Metformin inhibits NETs in diabetic patients (55). Metformin has also been shown to block mechanistic target of rapamycin (mTOR). Sirolimus, another mTOR inhibitor has been shown to block MERS-CoV infection in vitro and significantly improve ICU patient outcome (56, 57). Clinical trials investigating mTOR inhibitors against COVID-19 are currently recruiting participants. Metformin might have potential as a therapy for COVID-19.

Dimethyl fumarate (DMF) is a common therapy for adult patients with relapsing-remitting multiple sclerosis (58, 59). Studies suggest that DMF might have a similar mechanism of action as itaconate by activating NRF2 and target enzymes in glycolysis (60). DMF inhibits SARS-CoV-2 replication and replication of other viruses such as VACV, Zika virus, HSV1/2 in an interferon independent mechanism (32). DMF also suppresses inflammatory responses seen in severe COVID-19 by both liberating NRF2 and inhibiting the NF-κB signalling pathway by preventing p65 nuclear translocation. This has been shown to inhibit the transcription of inflammatory cytokines IFNB1, CXCL10, and CCL5 gene expression and upregulation of anti-inflammatory genes HMOX1 (32) (61). Furthermore, activating NRF2 also blocks transmembrane protease serine 2 (TMPRSS2) expression, which is needed for ACE2-mediated cell penetration of SARS-CoV-2 via the spike protein (62, 63). Given that DMF is an approved medication further research on whether it reduces severity of COVID19 is warranted.

## Conclusion

Studies into how SARS-CoV-2 affects intracellular metabolism during infection reveals interesting biochemical features, including increased glycolysis and dysregulated oxidative phosphorylation in innate immune cells. Preventing these metabolic changes in immune cells such as macrophages which might yield new anti-viral and anti-inflammatory therapies. Itaconate might have potential antiviral and anti-inflammatory potential. The mounting of a robust innate immune response in the early phase of SARS-CoV-2 infection is important to reduce viral load. However, the ability to limit the viral-mediated metabolic reprogramming in host immune cells and other infected cells to reduce the excessive inflammatory response seen in the later phase of SARS-CoV-2 infection is an interesting prospect the treatment of individuals who progress to severe COVID-19. The anti-viral and immunomodulatory properties of itaconate derivatives and DMF may give us two for the price of one; inhibition of viral replication and suppression of inflammation. Given these features, further studies with these metabolic modulators are well justified.

1. Bourdon M, Manet C, Montagutelli X. Host genetic susceptibility to viral infections: the role of type I interferon induction. Genes Immun. 2020;21(6-8):365-79.
2. O'Neill LA, Kishton RJ, Rathmell J. A guide to immunometabolism for immunologists. Nat Rev Immunol. 2016;16(9):553-65.
3. Lauterbach MA, Hanke JE, Serefidou M, Mangan MSJ, Kolbe CC, Hess T, et al. Toll-like Receptor Signaling Rewires Macrophage Metabolism and Promotes Histone Acetylation via ATP-Citrate Lyase. Immunity. 2019;51(6):997-1011.e7.
4. Selak MA, Armour SM, MacKenzie ED, Boulahbel H, Watson DG, Mansfield KD, et al. Succinate links TCA cycle dysfunction to oncogenesis by inhibiting HIF-alpha prolyl hydroxylase. Cancer Cell. 2005;7(1):77-85.
5. Mills EL, Kelly B, Logan A, Costa ASH, Varma M, Bryant CE, et al. Succinate Dehydrogenase Supports Metabolic Repurposing of Mitochondria to Drive Inflammatory Macrophages. Cell. 2016;167(2):457-70.e13.
6. Rouse BT, Sehrawat S. Immunity and immunopathology to viruses: what decides the outcome? Nat Rev Immunol. 2010;10(7):514-26.
7. Goodwin CM, Xu S, Munger J. Stealing the Keys to the Kitchen: Viral Manipulation of the Host Cell Metabolic Network. Trends Microbiol. 2015;23(12):789-98.
8. Ramière C, Rodriguez J, Enache LS, Lotteau V, André P, Diaz O. Activity of hexokinase is increased by its interaction with hepatitis C virus protein NS5A. J Virol. 2014;88(6):3246-54.
9. Bajwa G, DeBerardinis RJ, Shao B, Hall B, Farrar JD, Gill MA. Cutting Edge: Critical Role of Glycolysis in Human Plasmacytoid Dendritic Cell Antiviral Responses. J Immunol. 2016;196(5):2004-9.
10. Reyes A, Corrales N, Gálvez NMS, Bueno SM, Kalergis AM, González PA. Contribution of hypoxia inducible factor-1 during viral infections. Virulence. 2020;11(1):1482-500.
11. Keshavarz M, Solaymani-Mohammadi F, Namdari H, Arjeini Y, Mousavi MJ, Rezaei F. Metabolic host response and therapeutic approaches to influenza infection. Cell Mol Biol Lett. 2020;25:15.
12. Hadjadj J, Yatim N, Barnabei L, Corneau A, Boussier J, Smith N, et al. Impaired type I interferon activity and inflammatory responses in severe COVID-19 patients. Science. 2020;369(6504):718-24.
13. Merad M, Martin JC. Pathological inflammation in patients with COVID-19: a key role for monocytes and macrophages. Nat Rev Immunol. 2020;20(6):355-62.
14. Codo AC, Davanzo GG, Monteiro LB, de Souza GF, Muraro SP, Virgilio-da-Silva JV, et al. Elevated Glucose Levels Favor SARS-CoV-2 Infection and Monocyte Response through a HIF-1α/Glycolysis-Dependent Axis. Cell Metab. 2020;32(3):437-46.e5.
15. Zhou F, Yu T, Du R, Fan G, Liu Y, Liu Z, et al. Clinical course and risk factors for mortality of adult inpatients with COVID-19 in Wuhan, China: a retrospective cohort study. Lancet. 2020;395(10229):1054-62.
16. Song JW, Lam SM, Fan X, Cao WJ, Wang SY, Tian H, et al. Omics-Driven Systems Interrogation of Metabolic Dysregulation in COVID-19 Pathogenesis. Cell Metab. 2020;32(2):188-202.e5.
17. Strelko CL, Lu W, Dufort FJ, Seyfried TN, Chiles TC, Rabinowitz JD, et al. Itaconic acid is a mammalian metabolite induced during macrophage activation. J Am Chem Soc. 2011;133(41):16386-9.
18. Michelucci A, Cordes T, Ghelfi J, Pailot A, Reiling N, Goldmann O, et al. Immune-responsive gene 1 protein links metabolism to immunity by catalyzing itaconic acid production. Proc Natl Acad Sci U S A. 2013;110(19):7820-5.

19. Lampropoulou V, Sergushichev A, Bambouskova M, Nair S, Vincent EE, Loginicheva E, et al. Itaconate Links Inhibition of Succinate Dehydrogenase with Macrophage Metabolic Remodeling and Regulation of Inflammation. Cell Metab. 2016;24(1):158-66.
20. Yu XH, Zhang DW, Zheng XL, Tang CK. Itaconate: an emerging determinant of inflammation in activated macrophages. Immunol Cell Biol. 2019;97(2):134-41.
21. Cordes T, Michelucci A, Hiller K. Itaconic Acid: The Surprising Role of an Industrial Compound as a Mammalian Antimicrobial Metabolite. Annu Rev Nutr. 2015;35:451-73.
22. Swain A, Bambouskova M, Kim H, Andhey PS, Duncan D, Auclair K, et al. Comparative evaluation of itaconate and its derivatives reveals divergent inflammasome and type I interferon regulation in macrophages. Nat Metab. 2020;2(7):594-602.
23. Hooftman A, Angiari S, Hester S, Corcoran SE, Runtsch MC, Ling C, et al. The Immunomodulatory Metabolite Itaconate Modifies NLRP3 and Inhibits Inflammasome Activation. Cell Metab. 2020;32(3):468-78.e7.
24. Rodrigues TS, de Sá KSG, Ishimoto AY, Becerra A, Oliveira S, Almeida L, et al. Inflammasomes are activated in response to SARS-CoV-2 infection and are associated with COVID-19 severity in patients. J Exp Med. 2021;218(3).
25. Gurung P, Malireddi RK, Anand PK, Demon D, Vande Walle L, Liu Z, et al. Toll or interleukin-1 receptor (TIR) domain-containing adaptor inducing interferon-β (TRIF)-mediated caspase-11 protease production integrates Toll-like receptor 4 (TLR4) protein- and Nlrp3 inflammasome-mediated host defense against enteropathogens. J Biol Chem. 2012;287(41):34474-83.
26. Daniels BP, Kofman SB, Smith JR, Norris GT, Snyder AG, Kolb JP, et al. The Nucleotide Sensor ZBP1 and Kinase RIPK3 Induce the Enzyme IRG1 to Promote an Antiviral Metabolic State in Neurons. Immunity. 2019;50(1):64-76.e4.
27. Cho H, Proll SC, Szretter KJ, Katze MG, Gale M, Diamond MS. Differential innate immune response programs in neuronal subtypes determine susceptibility to infection in the brain by positive-stranded RNA viruses. Nat Med. 2013;19(4):458-64.
28. Mills EL, Ryan DG, Prag HA, Dikovskaya D, Menon D, Zaslona Z, et al. Itaconate is an anti-inflammatory metabolite that activates Nrf2 via alkylation of KEAP1. Nature. 2018;556(7699):113-7.
29. Bambouskova M, Gorvel L, Lampropoulou V, Sergushichev A, Loginicheva E, Johnson K, et al. Electrophilic properties of itaconate and derivatives regulate the IκBζ-ATF3 inflammatory axis. Nature. 2018;556(7702):501-4.
30. Bambouskova M, Potuckova L, Paulenda T, Kerndl M, Mogilenko DA, Lizotte K, et al. Itaconate confers tolerance to late NLRP3 inflammasome activation. Cell Rep. 2021;34(10):108756.
31. Kobayashi EH, Suzuki T, Funayama R, Nagashima T, Hayashi M, Sekine H, et al. Nrf2 suppresses macrophage inflammatory response by blocking proinflammatory cytokine transcription. Nat Commun. 2016;7:11624.
32. Olagnier D, Farahani E, Thyrsted J, Blay-Cadanet J, Herengt A, Idorn M, et al. SARS-CoV2-mediated suppression of NRF2-signaling reveals potent antiviral and anti-inflammatory activity of 4-octyl-itaconate and dimethyl fumarate. Nat Commun. 2020;11(1):4938.
33. Olagnier D, Brandtoft AM, Gunderstofte C, Villadsen NL, Krapp C, Thielke AL, et al. Nrf2 negatively regulates STING indicating a link between antiviral sensing and metabolic reprogramming. Nat Commun. 2018;9(1):3506.
34. Sethy B, Hsieh CF, Lin TJ, Hu PY, Chen YL, Lin CY, et al. Design, Synthesis, and Biological Evaluation of Itaconic Acid Derivatives as Potential Anti-Influenza Agents. J Med Chem. 2019;62(5):2390-403.

35. Zhang L, Wei L, Jiang D, Wang J, Cong X, Fei R. SARS-CoV nucleocapsid protein induced apoptosis of COS-1 mediated by the mitochondrial pathway. Artif Cells Blood Substit Immobil Biotechnol. 2007;35(2):237-53.
36. Guzzi PH, Mercatelli D, Ceraolo C, Giorgi FM. Master Regulator Analysis of the SARS-CoV-2/Human Interactome. J Clin Med. 2020;9(4).
37. Singh K, Chen YC, Judy JT, Seifuddin F, Tunc I, Pirooznia M. Network Analysis and Transcriptome Profiling Identify Autophagic and Mitochondrial Dysfunctions in SARS-CoV-2 Infection. bioRxiv. 2020.
38. Murphy MP, Smith RA. Targeting antioxidants to mitochondria by conjugation to lipophilic cations. Annu Rev Pharmacol Toxicol. 2007;47:629-56.
39. Dashdorj A, Jyothi KR, Lim S, Jo A, Nguyen MN, Ha J, et al. Mitochondria-targeted antioxidant MitoQ ameliorates experimental mouse colitis by suppressing NLRP3 inflammasome-mediated inflammatory cytokines. BMC Med. 2013;11:178.
40. Kelly B, Tannahill GM, Murphy MP, O'Neill LA. Metformin Inhibits the Production of Reactive Oxygen Species from NADH:Ubiquinone Oxidoreductase to Limit Induction of Interleukin-1β (IL-1β) and Boosts Interleukin-10 (IL-10) in Lipopolysaccharide (LPS)-activated Macrophages. J Biol Chem. 2015;290(33):20348-59.
41. Hu M, Bogoyevitch MA, Jans DA. Subversion of Host Cell Mitochondria by RSV to Favor Virus Production is Dependent on Inhibition of Mitochondrial Complex I and ROS Generation. Cells. 2019;8(11).
42. Corcoran SE, O'Neill LA. HIF1α and metabolic reprogramming in inflammation. J Clin Invest. 2016;126(10):3699-707.
43. McElvaney OJ, McEvoy NL, McElvaney OF, Carroll TP, Murphy MP, Dunlea DM, et al. Characterization of the Inflammatory Response to Severe COVID-19 Illness. Am J Respir Crit Care Med. 2020;202(6):812-21.
44. Palsson-McDermott EM, Curtis AM, Goel G, Lauterbach MA, Sheedy FJ, Gleeson LE, et al. Pyruvate kinase M2 regulates Hif-1α activity and IL-1β induction and is a critical determinant of the warburg effect in LPS-activated macrophages. Cell Metab. 2015;21(1):65-80.
45. Bouhlel MA, Derudas B, Rigamonti E, Dièvart R, Brozek J, Haulon S, et al. PPARgamma activation primes human monocytes into alternative M2 macrophages with anti-inflammatory properties. Cell Metab. 2007;6(2):137-43.
46. Neri T, Armani C, Pegoli A, Cordazzo C, Carmazzi Y, Brunelleschi S, et al. Role of NF-kappaB and PPAR-gamma in lung inflammation induced by monocyte-derived microparticles. Eur Respir J. 2011;37(6):1494-502.
47. Ghaffari S, Roshanravan N, Tutunchi H, Ostadrahimi A, Pouraghaei M, Kafil B. Oleoylethanolamide, A Bioactive Lipid Amide, as A Promising Treatment Strategy for Coronavirus/COVID-19. Arch Med Res. 2020;51(5):464-7.
48. Desterke C, Turhan AG, Bennaceur-Griscelli A, Griscelli F. PPARγ Cistrome Repression during Activation of Lung Monocyte-Macrophages in Severe COVID-19. iScience. 2020;23(10):101611.
49. Bailey CJ. Metformin: historical overview. Diabetologia. 2017;60(9):1566-76.
50. Singh S, Singh PK, Suhail H, Arumugaswami V, Pellett PE, Giri S, et al. AMP-Activated Protein Kinase Restricts Zika Virus Replication in Endothelial Cells by Potentiating Innate Antiviral Responses and Inhibiting Glycolysis. J Immunol. 2020;204(7):1810-24.
51. Luo P, Qiu L, Liu Y, Liu XL, Zheng JL, Xue HY, et al. Metformin Treatment Was Associated with Decreased Mortality in COVID-19 Patients with Diabetes in a Retrospective Analysis. Am J Trop Med Hyg. 2020;103(1):69-72.

52. Iwasaki M, Saito J, Zhao H, Sakamoto A, Hirota K, Ma D. Inflammation Triggered by SARS-CoV-2 and ACE2 Augment Drives Multiple Organ Failure of Severe COVID-19: Molecular Mechanisms and Implications. Inflammation. 2021;44(1):13-34.
53. Liu J, Li X, Lu Q, Ren D, Sun X, Rousselle T, et al. AMPK: a balancer of the renin-angiotensin system. Biosci Rep. 2019;39(9).
54. Barnes BJ, Adrover JM, Baxter-Stoltzfus A, Borczuk A, Cools-Lartigue J, Crawford JM, et al. Targeting potential drivers of COVID-19: Neutrophil extracellular traps. J Exp Med. 2020;217(6).
55. Carestia A, Frechtel G, Cerrone G, Linari MA, Gonzalez CD, Casais P, et al. NETosis before and after Hyperglycemic Control in Type 2 Diabetes Mellitus Patients. PLoS One. 2016;11(12):e0168647.
56. Kindrachuk J, Ork B, Hart BJ, Mazur S, Holbrook MR, Frieman MB, et al. Antiviral potential of ERK/MAPK and PI3K/AKT/mTOR signaling modulation for Middle East respiratory syndrome coronavirus infection as identified by temporal kinome analysis. Antimicrob Agents Chemother. 2015;59(2):1088-99.
57. Wang CH, Chung FT, Lin SM, Huang SY, Chou CL, Lee KY, et al. Adjuvant treatment with a mammalian target of rapamycin inhibitor, sirolimus, and steroids improves outcomes in patients with severe H1N1 pneumonia and acute respiratory failure. Crit Care Med. 2014;42(2):313-21.
58. Fox RJ, Kita M, Cohan SL, Henson LJ, Zambrano J, Scannevin RH, et al. BG-12 (dimethyl fumarate): a review of mechanism of action, efficacy, and safety. Curr Med Res Opin. 2014;30(2):251-62.
59. Gold R, Kappos L, Arnold DL, Bar-Or A, Giovannoni G, Selmaj K, et al. Placebo-controlled phase 3 study of oral BG-12 for relapsing multiple sclerosis. N Engl J Med. 2012;367(12):1098-107.
60. Kornberg MD, Bhargava P, Kim PM, Putluri V, Snowman AM, Putluri N, et al. Dimethyl fumarate targets GAPDH and aerobic glycolysis to modulate immunity. Science. 2018;360(6387):449-53.
61. Kastrati I, Siklos MI, Calderon-Gierszal EL, El-Shennawy L, Georgieva G, Thayer EN, et al. Dimethyl Fumarate Inhibits the Nuclear Factor κB Pathway in Breast Cancer Cells by Covalent Modification of p65 Protein. J Biol Chem. 2016;291(7):3639-47.
62. Schultz MA, Hagan SS, Datta A, Zhang Y, Freeman ML, Sikka SC, et al. Nrf1 and Nrf2 transcription factors regulate androgen receptor transactivation in prostate cancer cells. PLoS One. 2014;9(1):e87204.
63. Hoffmann M, Kleine-Weber H, Schroeder S, Krüger N, Herrler T, Erichsen S, et al. SARS-CoV-2 Cell Entry Depends on ACE2 and TMPRSS2 and Is Blocked by a Clinically Proven Protease Inhibitor. Cell. 2020;181(2):271-80.e8.

| | SARS-CoV-2 | WNV | ZIKV | HSV1/2 | VACV | Influenza A |
|---|---|---|---|---|---|---|
| Itaconate | ND | (-) | (-) | ND | ND | (-) |
| 4-OI | (-) | ND | (-) | (-) | (-) | ND |
| DMF | (-) | ND | (-) | (-) | (-) | ND |
| Metformin | (-) | ND | (-) | ND | ND | (-) |

Table 1. Antiviral immunomodulators. Viral inhibition is annotated with "(-)", not determined is annotated with "ND".

Figure 1. Schematic of SARS-CoV-2 infection in the lung.SARS-CoV-2 infection of airway epithelial cells and macrophages causes an upregulation of glycolysis and fatty acid synthesis (FAS), to enable viral replication and release of SARS-CoV-2. Viral sensing by PRRs causes interferon production, while increased glycolysis and FAS induces inflammatory cytokine production. Macrophages can be infected by SARS-CoV-2 in the same mechanism or can be polarised by inflammatory cytokines produced by airway epithelial cells. M1-like polarisation of macrophages causes the production of inflammatory cytokines that contribute to the immunopathology seen in COVID-19. Created with Biorender.com

Figure 2. Metabolic modulators to treat COVID-19. 4-OI and DMF activate NRF2 which inhibit inflammatory cytokines. NRF2 activation prevents IRF-3 signaling, inhibiting interferon responses. 4-OI and DMF prevent TMPRSS2 expression which may prevent SARS-CoV-2 cell entry. MitoQ blocks SARS-CoV-2 replication. MitoQ increases autophagy and reduces mtROS to reduce inflammatory cytokines expression. TEPP-46 maintains PKM2 in an inactive conformation, preventing activation of HIF1$\alpha$ and associated inflammatory cytokines. Metformin phosphorylates ACE2 Ser680 which might prevent viral entry. Metformin upregulates ACE2, preventing RAS-mediated inflammation. Metformin inhibits inflammatory cytokine IL1β and upregulates anti-inflammatory cytokine IL-10. Created with Biorender.com

Data Availability statement: Not applicable

Competing interests: The authors declare no competing interests

Funding statement:  The O'Neill laboratory acknowledges the following grant support: European Research Council Metabinate (834370), Science Foundation Ireland (12/IA/ 1531), and The Wellcome Trust (205455).

Acknowledgements: Not applicable

Author contributions: S.O'C and L.A.J.O'N. wrote the manuscript

Reviewer acknowledgement: The Editor-in-Chief, Tim Elliott, and handling editor, Marianne Boes, would like to thank the following reviewers, Pedro Moraes Vieira and Bart Everts, for their contribution to the publication of this article.

Figure 1

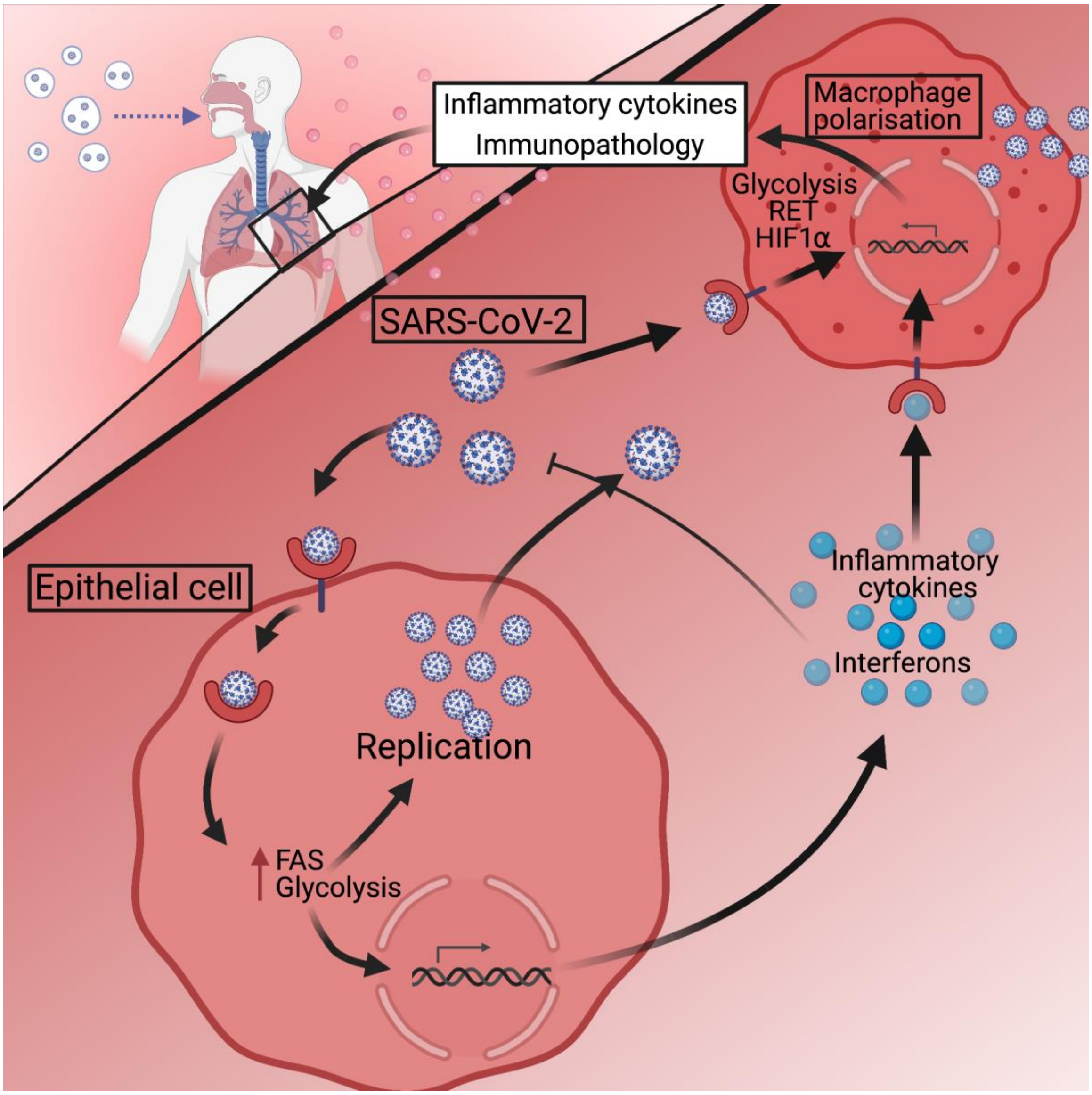

Inflammatory cytokines
Immunopathology
Macrophage polarisation
Glycolysis
RET
HIF1α
SARS-CoV-2
Epithelial cell
Replication
FAS
Glycolysis
Inflammatory cytokines
Interferons

Figure 2

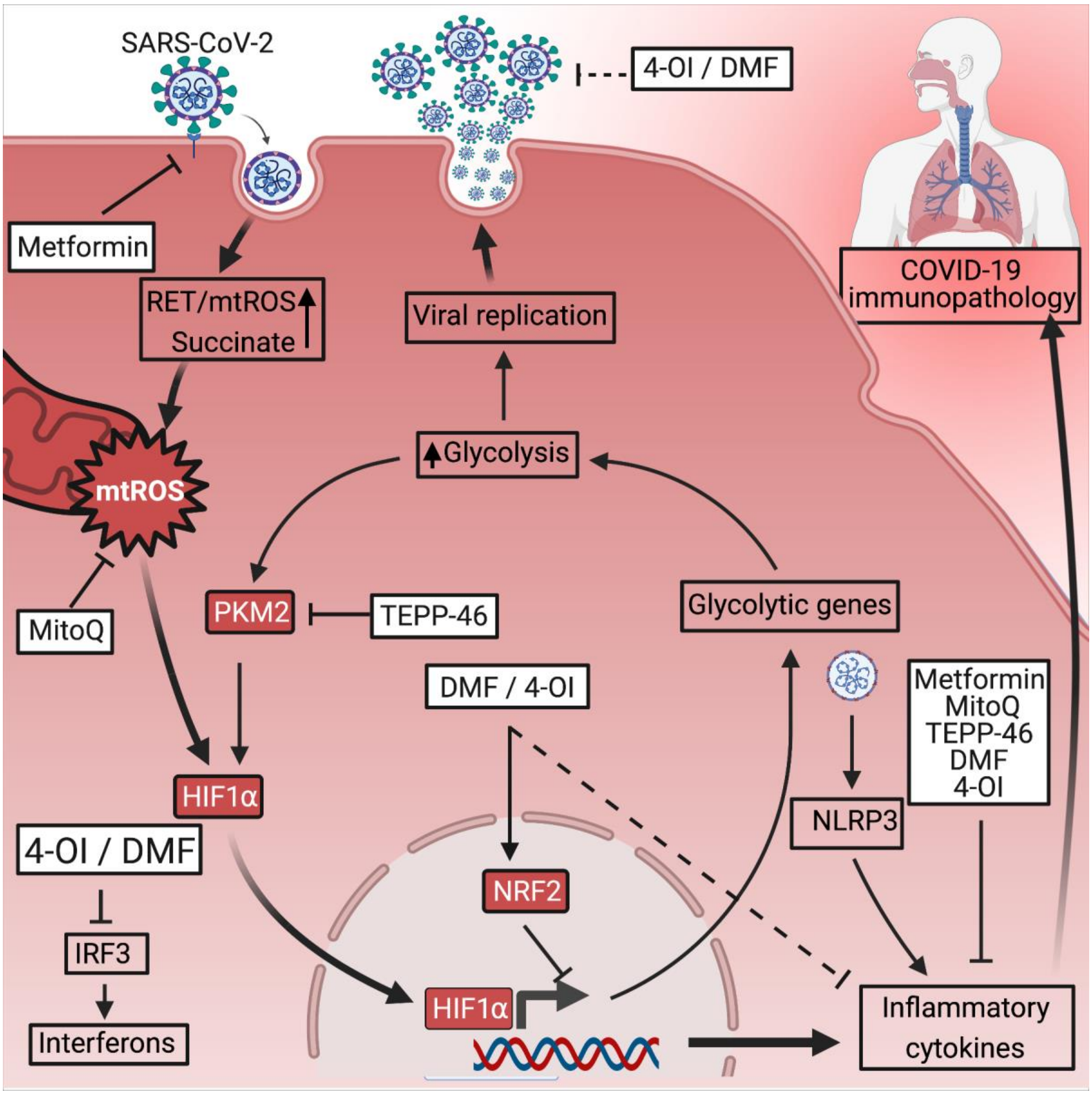

SARS-CoV-2
4-OI / DMF
Metformin
RET/mtROS
Succinate
Viral replication
COVID-19 immunopathology
mtROS
Glycolysis
MitoQ
PKM2
TEPP-46
Glycolytic genes
DMF / 4-OI
Metformin
MitoQ
TEPP-46
DMF
4-OI
HIF1α
4-OI / DMF
NLRP3
NRF2
IRF3
HIF1α
Inflammatory cytokines
Interferons
